# Burden of asthma with elevated blood eosinophil levels

**DOI:** 10.1186/s12890-016-0263-8

**Published:** 2016-07-13

**Authors:** Julian Casciano, Jerry A. Krishnan, Mary Buatti Small, Philip O. Buck, Gokul Gopalan, Chenghui Li, Robert Kemp, Zenobia Dotiwala

**Affiliations:** eMAX Health LLC, 445 Hamilton avenue, 11th floor, White Plains, NY 10601 USA; Division of Pulmonary, Critical Care, Sleep, and Allergy, University of Illinois at Chicago, Chicago, IL USA; Teva, Frazer, PA USA; University of Arkansas for Medical Sciences, Little Rock, AR USA

**Keywords:** Severe asthma, Economic burden, Elevated eosinophils

## Abstract

**Background:**

Asthma is a common chronic condition with an economic burden of almost $56 billion annually in the US. Biologic markers like blood eosinophils, that help predict the risk of exacerbation could help guide more optimal treatment plans and reduce cost. The purpose of this study was to determine whether healthcare resource use and expenditures vary by eosinophil level among patients with asthma.

**Methods:**

Patients with a diagnosis of asthma defined by ICD-9-CM code 493.xx between January 2004 and July 2011 were extracted from EMRClaims + database (eMAX Health, White Plains NY). Patients were classified as mild, moderate, or severe by medication use following diagnosis, based on recommendations of National Institutes of Health Expert Panel Report 3. Patients were classified as those with elevated eosinophils (≥400 cells/μL) and normal eosinophil level (<400 cells/μL). Patients were followed for resource use, defined as hospitalizations, ER visits and outpatient visit and associated costs were calculated to assess whether an economic difference exists between eosinophil groups. Non-parametric tests were used to compare resource use and associated cost between elevated and normal eosinophil groups. Multivariate modeling was performed to assess the contribution of eosinophil level on the likelihood of study outcomes among patients with severe asthma.

**Results:**

Among the 2,164 patients meeting eligibility criteria, 1,144 had severity designations. Of these, 179(16 %) of patients had severe asthma of which 20 % (*n* = 35) had elevated eosinophils. Seventeen percent of patients with elevated eosinophils were admitted to the hospital during the follow-up period, significantly greater than patients with normal eosinophil levels (12 %; *p* = 0.011). Overall, compared to patients with normal eosinophil levels (*n* = 1734), patients with elevated eosinophil levels (*n* = 430) had significantly greater mean annual hospital admissions (0.51 vs. 0.21/year, *p* = 0.006) and hospital costs (2,536 vs. $1,091, *p* = 0.011). Logistic regressions showed that elevated eosinophil level was associated with 5.14 times increased odds of all cause admissions (95 % CI:1.76–14.99, *p* = 0.003) and 4.07 times increased odds of asthma related admissions (95 % CI: 1.26–13.12, *p* = 0.019).

**Conclusion:**

Eosinophil elevation was associated with greater healthcare resource use in patients with asthma.

## Background

Asthma has been reported to affect as many as 26 million US adults with 51 % reporting asthma exacerbations in 2011 [1]. In 2010 alone, there were over 400,000 persons hospitalized in the US for asthma exacerbations [1]. In 2010, US patients with asthma logged over 10 million physician office visits and over 2 million ER visits [1]. The overall annual economic burden caused by asthma is as high as $564 billion, with 89 % in direct healthcare cost [1].

In the overall burden caused by asthma, severe asthma, specifically, results in a greater number of exacerbations, healthcare utilization, and expenditures [2]. Exacerbations among moderate and severe asthma patients increase the frequency of hospital admissions by nearly 50 %, and emergency department visits by 100 %, compared to moderate and severe asthma patients without exacerbations. This added healthcare utilization contributes to about $4000 increase in per patient cost annually [3].

Patients with eosinophilic asthma (EA) are of particular concern since disease severity has been shown to correlate with the level of eosinophils detected in blood and the bronchoalveolar fluid [4]. When elevated, eosinophils cause an immune-modulatory response which includes airway inflammation and hyper responsiveness, damage to epithelial lining, and excess secretion of mucus [5]. Sputum eosinophil levels are helpful in characterizing airway inflammation, predicting response to corticosteroid treatment, and identifying patients at risk of exacerbations [5, 6]. However, sputum eosinophil measurements requires specialized training to collect, process, and analyze and is not generally available in clinical settings. Researchers have reported a potential association between fractional exhaled nitric oxide (FeNO) levels and eosinophilic airway inflammation which may be helpful as a non-invasive marker for EA in clinical practice [7]. We have presented data showing an association between peripheral blood eosinophilia and moderate-to-severe asthma severity defined by Expert Panel-3 guidelines, given that Complete Blood Count (CBC) with Differential tests are routinely ordered for asthma patients [8]. However, it is not clear whether elevated serum eosinophil level is associated with a greater likelihood of hospitalization and elevated cost in asthma patients. The objective of this study was to understand the relationship between eosinophil level and healthcare utilization and expenditures in patients with asthma, as well the subset with severe asthma. Demonstrated predictive value of eosinophils in asthma control and system cost would support the utility of more focused identification and management of this patient phenotype.

## Methods

### Study design and data source

We conducted a retrospective cohort analysis of US asthma patients between January 2004 and July 2011. Patients with a primary or secondary diagnosis of asthma were followed to assess their resource use and cost. Data was extracted from EMRClaims+, an integrated health services database of patients located in the Midwest region of the United States. The database includes administrative insurance claims from a managed care plan of approximately 675,000 lives linked to an overlapping healthcare provider database of electronic medical records data (EMR), including laboratory values, and provider billing files. The database also tracks commercially insured lives through provider-aligned patient panels, managed care membership files and a Master Patient Index.

### Study population

All patients with at least two encounters in the inpatient (to confirm asthma diagnosis), emergency room (ER), or outpatient (OP) setting with an International Classification of Diseases-9- Clinical Modification [ICD-9-CM] code 493.xx as the primary or secondary diagnosis, were selected. The date of the first asthma diagnosis during the study period was defined as the index diagnosis date. Patients less than 12 years of age at the time of index diagnosis were excluded. Patients were required to be continuously enrolled for a period of at least 13 months after the index date, consisting of a 12 month ‘assessment period’ to establish severity classifications based on medication use and to record eosinophil test results, and a follow-up of 1 to 12 months after this assessment period during which outcomes were assessed. Patients were excluded if during the assessment period: 1) they had no eosinophil tests; 2) all eosinophil tests were conducted while on systematic steroids (defined as eosinophil test dates overlapping with the periods of potential systemic steroid use based on date of prescription fill, days of supply of medication plus a 14 day washout period) and the results were all under 400 cells/μL; this exclusion criteria was to avoid including patients with lower eosinophil values due to the effect of systemic steroids [9]; or 3) if they had diagnoses of confounding disease states of COPD, emphysema, Churg Strauss syndrome, Wegener’s granulomatosis, hypereosinophilic syndrome, pulmonary fibrosis, allergic bronchopulmonary aspergillosis and lung cancer (ICD-9-CM codes: 491.xx-492.xx, 494.xx-496.xx, 277.x, 162.x, 446.4, 288.3, 516.31, 515, 518.6).

### Study Measures

The National Heart, Lung, and Blood Institute’s Expert Panel Report 3: Guidelines for the Diagnosis and Management of Asthma (EPR-3) criteria were adapted to classify patients into mild, moderate, and severe disease based on the pattern of asthma medications (Table 1). For example, patients with use of high dose inhaled corticosteroids (ICS) and long acting beta agonists (LABA) or a combination of the two and oral corticosteroids, at any point during the assessment period, were classified as having severe asthma. Patients with only low dose ICS use at any time during the entire assessment period were classified as those with mild asthma. Given the lack of ‘impairment’ measures such as night time awakenings we restricted the definition of ‘severe’ patients to those taking high-dose ICS as described in Table 1. Patients were classified by eosinophil level as “elevated” if at least one eosinophil test result in the assessment period was ≥ 400 cells/μL, or “normal” if none of the test results of a patient were ≥ 400 cells/μL. We selected this threshold based on results of research reported in the literature that show an association between eosinophil elevation and asthma severity at a threshold of ≥400 cells/μL [8, 10–12]. Consistent with data we have previously reported [8], Price and colleagues (2015) assessed the effect of eosinophils at lower cut-off values (≥200, ≥300 cells/μL) and found very weak to no associations with future exacerbations [10]. Patient demographics (age, gender, and race/ethnicity) were recorded. Patients’ comorbidity burden was measured using the Charlson Comorbidity Index (CCI).

**Table 1:** Definition of severity levels based on medication use

Severity level	Medications
Mild	•Low dose ICS or
	•Cromolyn, LTRA, nedocromil, or theophylline
Moderate	•Low-dose ICS + LABA OR Medium-dose ICS OR Medium-dose ICS + LABA, or
	•Low-dose ICS + either LeukoTriene Receptor Antagonist (LTRA),Theophylline, or Zileuton, or
	•Medium-dose ICS + either LTRA, Theophylline, or Zileuton
Severe	•High-dose ICS + LABA OR High-dose ICS + LABA + oral corticosteroid,or
	•High-dose ICS + LABA + _Omalizumab, or High-dose ICS + LABA + oral corticosteroid + Omalizumab

Resource use, defined as hospital admissions, emergency room (ER) visits and outpatient visits, and costs associated with resource were estimated during the post-assessment follow-up period. There were two sources of cost data in this database: encounters from Plan-owned sites, and external claims. For external claims, the claim amounts were included as costs. Plan-owned sites reported charges (since claims are not paid) from the administrative charge master. These charges were reduced to costs by multiplying the reported charges by a cost-to-charge ratio factor of 0.33 (derived from 2,158 asthma encounters). This factor was calculated using the Premier hospital database [13, 14], by comparing actual costs to reported charges from 95 different hospitals in Midwestern United States across nearly 13,000 encounters for patients diagnosed with asthma, with 2,158 asthma-related encounters (primary diagnosis). Frequency of ER visits, outpatient visits, and hospital admissions were also recorded. Annualized resource utilization and cost were also calculated by multiplying per patient per month (PPPM) values by 12.

### Data analysis

The PPPM resource utilization as well as mean costs among patients with asthma, identified in this study, were compared between those with normal eosinophil level vs. elevated eosinophil level. The statistical significance of the mean differences was evaluated using nonparametric tests. We used Chi-Square and Fisher Exact test to compare the proportion of patients who had each type of service use between patients with elevated eosinophils versus those with normal eosinophils. In the adjusted analysis, logistic regressions were used to assess the probability of resource consumption with eosinophil level as the key independent (predictor) variable. The models adjusted for other factors such as patient demographics, severity and CCI score. To account for differential follow-up time, weighted models based on the number of months in each patient’s post-assessment follow-up period were used. All data analyses were performed using SAS software, version 9.3 (SAS Institute Inc., Cary, North Carolina).

## Results

A total of 2,164 patients met the eligibility criteria, of whom 430 (16.5 %) had elevated eosinophil levels. A total of 1,144 were classified into the three severity groups with mild (40 %), moderate (44 %) and severe (16 %) asthma. Among severe patients, no significant differences in baseline patient characteristics were found between elevated and normal eosinophil levels (Table 2). The mean follow-up time for the patients with elevated and normal eosinophils in the overall sample (2,164) as well as among patients with severe asthma (179) was 11 months.

**Table 2:** Demographic and comorbidity distribution- Patients with Severe Asthma

Patient characteristics	Eosinophil level		P
	Elevated eosinophils (*n* = 35)	Normal eosinophils (*n* = 144)	
	N (%)	N (%)	
Gender			0.953
Female	24 (68.6)	98 (68.1)	
Race			0.608
White	20 (57.1)	74 (51.4)	
Black	5 (14.3)	14 (9.7)	
Hispanic	5 (14.3)	34 (23.6)	
Other/Unknown	5 (14.3)	22 (15.3)	
Age groups			0.165
12–17 years	2 (5.71)	5 (3.5)	
18–35 years	8 (22.86)	18(12.50)	
36–64 years	16 (45.71)	92(63.89)	
Greater than/equal to 65 years	9 (25.71)	29 (20.14)	
Top 5 Comorbidities			
Diabetes	7 (20.0)	24 (16.7)	0.640
Cancer/tumor	3 (8.6)	7 (4.9)	0.413
Congestive Heart Failure	4 (11.4)	7 (4.9)	0.229
Cerebrovascular disease	0 (0.0)	3 (2.1)	1
Renal disease	2 (5.71)	1 (0.7)	0.098

### All asthma patients (*n* = 2,164)

**Fig. 1:**
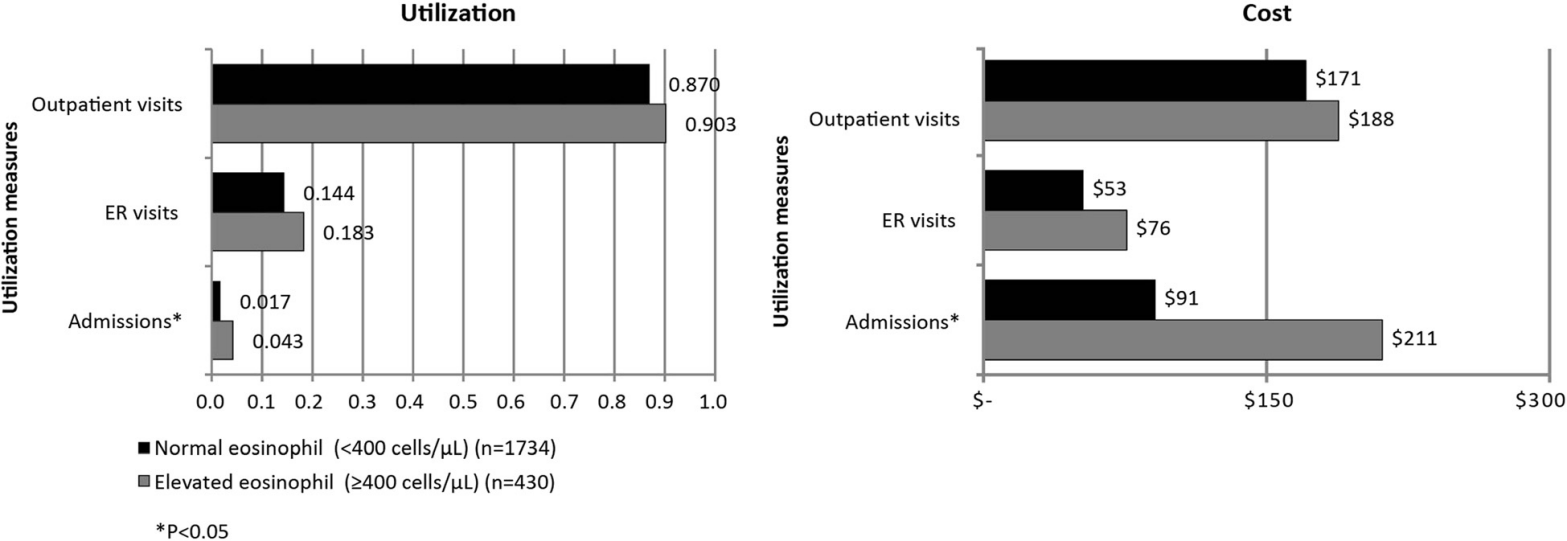
Mean Per Patient Per Month Utilization and Cost-Overall (*n* = 2,164)

Comparing mean unadjusted PPPM healthcare utilization between the elevated and normal eosinophil groups overall (*n* = 2,164) (Fig. 1) showed monthly all-cause hospitalizations were significantly greater in the elevated group (0.043 vs. 0.017, *p* = 0.006; annually 0.513 vs. 0.207, *p* = 0.006). Irrespective of severity, 17 % percent of patients with elevated eosinophils were admitted to the hospital during the follow-up period, significantly greater than patients with normal eosinophil levels (12 %; *p* = 0.011, Table 3). Mean monthly admission cost was 2.3 times greater for the elevated eosinophilia group (Fig. 1, annually $2,536 vs. $1,091, *p* = 0.011).

**Table 3:** Proportion of Patients with Resource Utilization during Follow-up

	Overall (*n* = 2,164)			Patients with severe asthma (*n* = 179)		
Resource use	Elevated eosinophils	Normal eosinophils	P value	Elevated eosinophils	Normal eosinophils	P value
	(*N* = 430)	(*N* = 1,734)		(*N* = 35)	(*N* = 144)	
Hospital admissions	73(17)	214(12)	0.011	10(29)	12(8)	0.001
ER visits	156(36)	622(36)	0.874	11(31)	47(33)	0.891
OP visits	373(87)	1536(89)	0.290	34(97)	134(93)	0.366

### Severe asthma patients (*n* = 179)

**Fig. 2:**
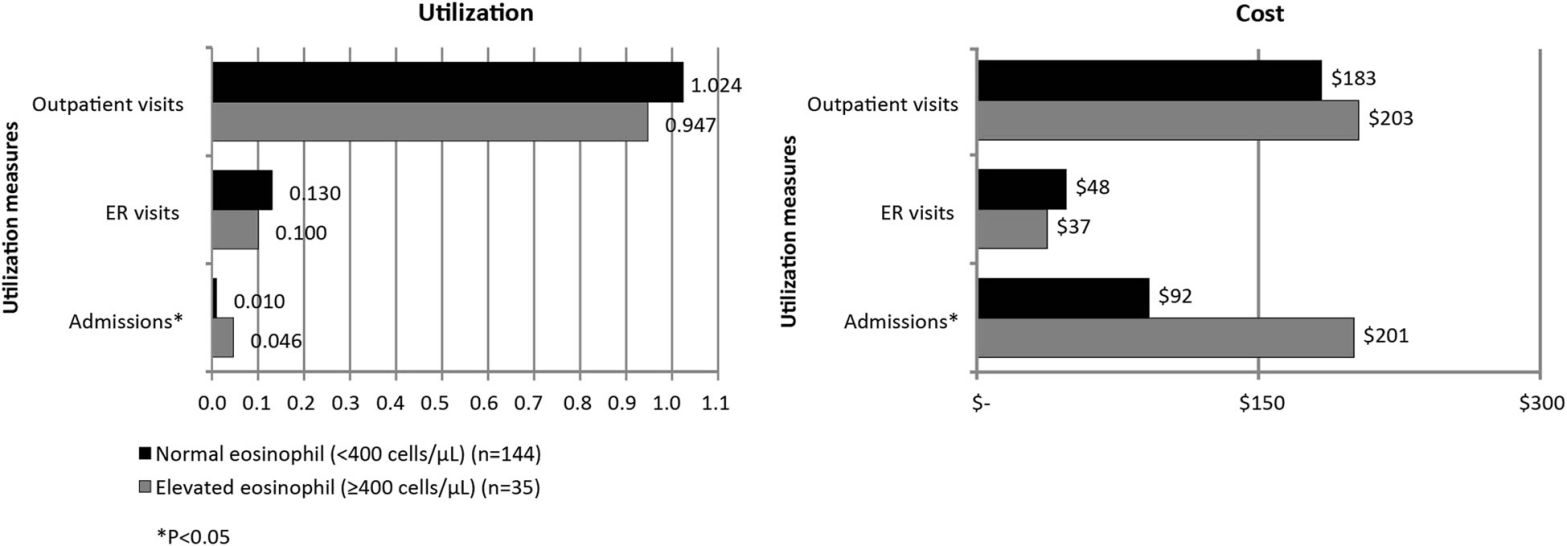
Mean Per Patient Per Month Utilization and Cost-Severe Asthma Patients (*n* = 179)

**Fig. 3:**
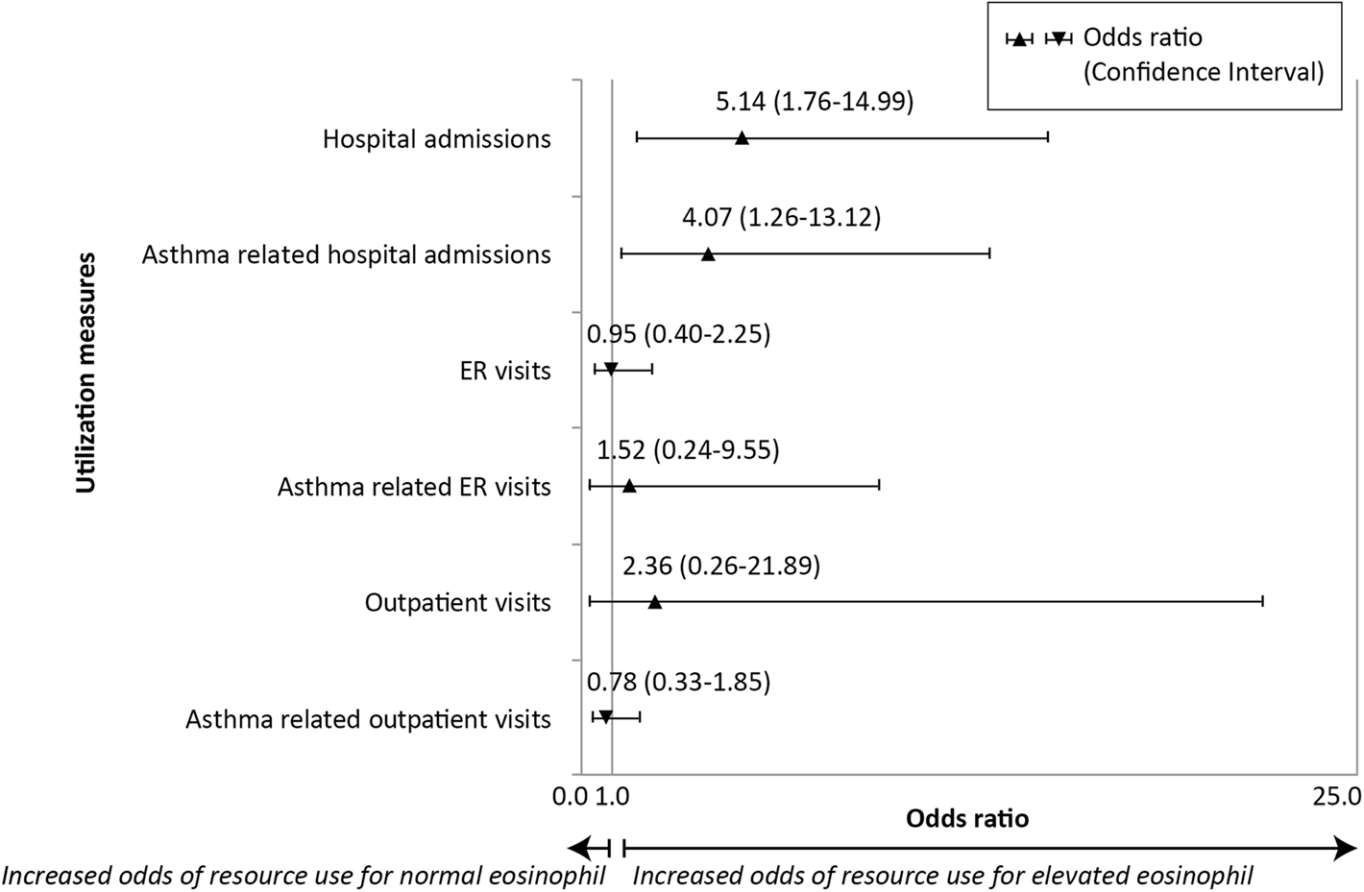
Magnitude of change in odds of incurring utilization among patients with severe asthma

Among patients with severe disease, both frequency and proportion of hospitalization were significantly greater for the elevated eosinophil group, with 4.6 times as many monthly hospital admissions per patient (0.046 vs. 0.010, *p* = 0.001, Fig. 2), and more than 3.5 times as many patients with at least one admission (29 % versus 8 %, Chi square *p* = 0.003, Table 3). No significant differences between the eosinophil groups for the number of outpatient and ER claims were detected. Mean monthly admission cost was significantly greater in the elevated eosinophil group for all-cause admissions (*p* = 0.002, Fig. 2). Annualized, the mean difference between elevated and normal eosinophil groups for severe asthma was estimated at $1,310 ($2,410 vs. $1,100, *p* = 0.002). Elevated eosinophil level increased the odds of all-cause admissions (OR = 5.14, 95 % CI: 1.76–14.99, *p* = 0.003, Fig. 3) as well as asthma related admissions (OR: 4.07, 95%CI: 1.26–13.12, *p* = 0.019) during follow-up.

## Discussion

Our study found that patients with severe asthma and elevated eosinophils at ≥400 cells/μL had a significantly greater number of unadjusted monthly all cause hospital admissions. Patients with elevated eosinophils were admitted to the hospital on average about once every 2 years, whereas patients with normal eosinophils were admitted about once every 5 years. Similarly, patients with severe asthma and elevated eosinophils were admitted to the hospital about once every 1.8 years, whereas patients with severe asthma and normal eosinophils were admitted only about once every 8.3 years. These findings suggest that serum eosinophil levels can be used to risk-stratify patients with asthma both overall and in the subset with severe asthma. We did not find significantly greater outpatient and ER visits for the elevated eosinophil group. This indicates a stronger association between eosinophil elevation and risk of severe exacerbations needing hospitalization, but not milder exacerbations that required only an ER visit or clinic visit. This difference could be explained by less successful therapeutic intervention with eosinophilia once in a state of exacerbation. The more costly hospital admissions were significantly greater, compared to normal eosinophil group. More frequent hospitalization was responsible for greater cost for the elevated eosinophil group. Adjusted analyses show that severe asthma patients with elevated eosinophils were significantly more likely to be admitted to the hospital compared to patients with normal eosinophils. To estimate the added cost to the U.S. of severe asthma patients with elevated eosinophils (versus normal eosinophils), we applied the proportion of severe disease and elevated eosinophils we have observed here (16 and 19 % respectively) to the 26 million asthma patients. Given the mean difference in admission cost for this segment (over $1,300), we estimate the added cost to the U.S. healthcare system attributable to this group is approximately $1.3 Billion.

## Limitations

We employed an approximation of EPR-3 recommendations regarding medication use to classify disease severity, rather than symptom control, lung function, or risk of exacerbations. Our method was an approximation because we did not identify the lowest dose of controller required for control, but instead relied on sporadic use. Our approach did not account for drug switching, potentially resulting in misclassification of an unknown fraction of patients, with lower severity than might actually be the case. Additional cohort bias was introduced because, for patients who were prescribed more medication than necessary to achieve asthma control, our approach misclassified patients with milder asthma as patients with more severe disease. Number of prescription claims, not actual prescription consumption, was utilized to assess medication use. However, medications were used to define severity and regardless of whether they actually took the medications, prescribers considered them appropriate given the patients’ level of severity. Patients might overuse SABA instead of being adjusted for long-term control medications which may lead to some misclassification. Despite rigorous inclusion/exclusion criteria for sample identification, oral corticosteroids can be used for diseases other than asthma which may misclassify patients as ‘severe’. All financial data are estimates of cost, not data from actual transaction. For a portion of encounters we used a cost-to-charge ratio because only charge data was reported. However, the cost-to-charge ratio was derived empirically from a large hospital sample for asthma patients in the same geography as our study. Additionally, due to case finding criteria, our regressions should be viewed in the context of a resulting smaller sample size for the severe group with elevated eosinophils.

## Conclusion

An elevated eosinophil level of at least 400 cells/μL was associated with greater resource use and estimated cost in overall asthma patients as well as those with severe asthma. Adjusted analysis showed that blood eosinophil elevation was significant in predicting probability of hospitalization among severe asthma patients on average, and the smaller group of severe asthma patients with elevated eosinophils drive healthcare expenditures. Our findings demonstrate the importance of further research to establish peripheral blood eosinophil elevation as a biomarker for disease control and overall healthcare expenditures.

## Abbreviations

CCI, Charlson comorbidity index; CI, confidence Interval; EMR, electronic medical record; EPR, expert panel report; ER, emergency room; ICD-9-CM, International Classification of Diseases-9- Clinical Modification; ICS, inhaled corticosteroid; LABA, long acting beta agonist; OP, outpatient; OR, odds ratio; PPPM, per patient per month
